# SureSelect targeted enrichment, a new cost effective method for the whole genome sequencing of *Candidatus* Liberibacter asiaticus

**DOI:** 10.1038/s41598-019-55144-4

**Published:** 2019-12-12

**Authors:** Weili Cai, Schyler Nunziata, John Rascoe, Michael J. Stulberg

**Affiliations:** 1 Science and Technology, Plant Protection and Quarantine, Animal and Plant Health Inspection Service, United States Department of Agriculture, Beltsville, Maryland United States of America; 2 Department of Entomology and Plant Pathology, North Carolina State University, Raleigh, North Carolina United States of America

**Keywords:** Pathogens, Plant biotechnology

## Abstract

Huanglongbing (HLB) is a worldwide deadly citrus disease caused by the phloem-limited bacteria ‘*Candidatus* Liberibacter asiaticus’ (*C*Las) vectored by Asian citrus psyllids. In order to effectively manage this disease, it is crucial to understand the relationship among the bacterial isolates from different geographical locations. Whole genome sequencing approaches will provide more precise molecular characterization of the diversity among populations. Due to the lack of *in vitro* culture, obtaining the whole genome sequence of *C*Las is still a challenge, especially for medium to low titer samples. Hundreds of millions of sequencing reads are needed to get good coverage of *C*Las from an HLB positive citrus sample. In order to overcome this limitation, we present here a new method, Agilent SureSelect ^XT HS^ target enrichment, which can specifically enrich *C*Las from a metagenomic sample while greatly reducing cost and increasing whole genome coverage of the pathogen. In this study, the *C*Las genome was successfully sequenced with 99.3% genome coverage and over 72X sequencing coverage from low titer tissue samples (equivalent to 28.52 Cq using Li 16 S qPCR). More importantly, this method also effectively captures regions of diversity in the *C*Las genome, which provides precise molecular characterization of different strains.

## Introduction

Huanglongbing (HLB), or citrus greening, is a devastating citrus disease caused by phloem-restricted gram-negative bacteria ‘*Candidatus* Liberibacter’ spp^1,2^. There are three α-proteobacteria associated with HLB: “*Candidatus* Liberibacter asiaticus”, “*Ca*. Liberibacter americanus” and “*Ca*. Liberibacter. africanus”^1,3^. ‘*Ca*. Liberibacter asiaticus’ (*C*Las) is the most widespread and is the only species associated with the disease in the United States (U.S.)^4^. *C*Las associated HLB was first found in Florida in early September, 2005^5^ and was vectored by the Asian citrus psyllid (*Diaphorina citri*), which had been introduced into Florida in the late 1990s. The disease has since been identified in multiple states (USDA APHIS Citrus Greening Quarantine map, https://www.aphis.usda.gov/plant_health/plant_pest_info/citrus_greening/downloads/pdf_files/nationalquarantinemap.pdf).

Effective disease managing efforts require a greater understanding of the causal agents, which can be achieved through whole genome sequencing. The genetic identity of strains found in new locations or with varying aggressiveness can help inform the effectiveness of quarantine programs and provide researchers with data to search for virulence-associated genetic elements. Identifying aggressive strains might impact future management practices if zero tolerance policies are no longer applicable. Providing strain identification can help inform pathogen dissemination.

Whole genome sequencing can provide precise molecular characterization of the diversity among *C*Las populations. Currently, conserved genomic loci, such as the 16S rRNA gene, are used to define the *C*Las species but lack the genetic variation to differentiate strains^6,7^. Population variation studies using PCR to amplify several genomic loci or short tandem repeats regions might not provide sufficiently high resolution to differentiate all strains from multiple locations^8–12^. A pan-genome comparative approach could provide enough genetic variation for high strain resolution, but sequencing *C*Las genomes has been historically difficult. The first *C*Las genome sequence was released in 2009, isolated from a single infected psyllid^13^, and in nearly 10 years since there have been only 14 additional *C*Las genomes deposited to NCBI (only five are complete). The released *C*Las genomes were obtained from either highly infected psyllids or citrus samples (equivalent to 18 to 23 Cq using Li 16S qPCR)^14–17^ because the whole genome sequence of *C*Las can only be obtained using metagenomic sequencing, due to the lack of *in vitro* culture. Such high pathogen titer samples are needed because a low percentage of sequencing reads belonging to *C*Las are present in a metagenomic sample, primarily because of large genome size difference between pathogen and host and relative low copy number of pathogen DNA. Hundreds of millions of sequencing reads are needed to get good *C*Las genome coverage from an infected citrus sample, making *C*Las genome sequencing challenging and costly^18^. Additionally, to study the impact of strain diversity in *C*Las epidemiology, it is important to include more geographic locations, and newly infected samples often carry a much lower pathogen titer than the successfully sequenced samples. Thus a targeted genome enrichment method may be useful and necessary.

Targeted genome enrichment specifically enriches sequences of interest within a heterogeneous mixture of DNA samples. For target selection, pre-designed probes are added to the mixed genomic DNA extracts and capture their complimentary DNA sequences through complimentary hybridization, allowing the uncaptured DNA to be removed during wash steps. With positive target selection, the probe-bound DNA is eluted and collected for further NGS application, and often has much higher target DNA concentration than the original input samples^19,20^. This method has been widely used to capture and enrich targeted DNA from complex biological samples, but is not commonly used to recover plant pathogens from a plant host background^21–23^.

In this study, we assess the ability of a target enrichment method, Agilent SureSelect ^XT HS^ (hereafter referred to as SureSelect), to enrich *C*Las genomic DNA from infected citrus genomic DNA, and in turn greatly reduce the cost and increase the coverage and reliability of whole genome sequencing.

## Results

### Genome alignment and target enrichment

**Figure 1:**
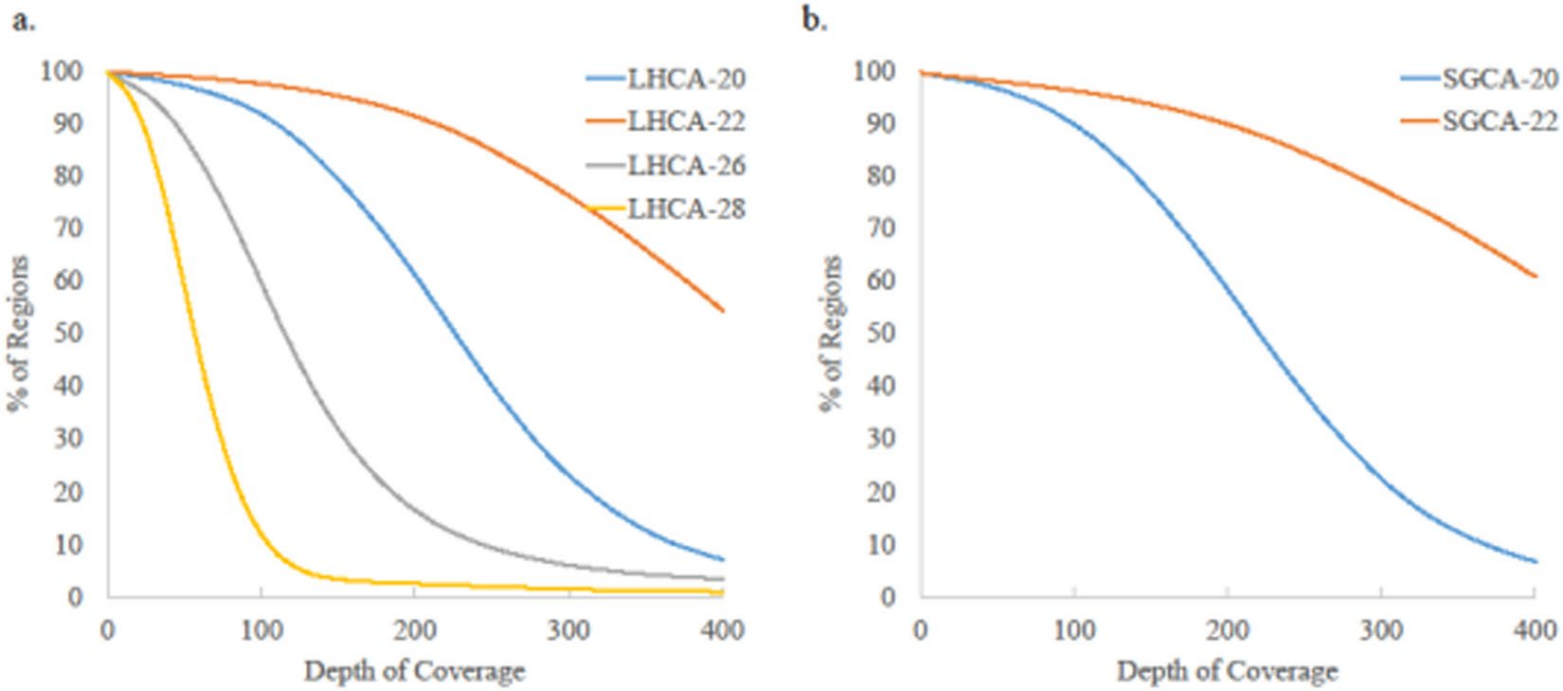
Percentage of bases covered across fixed depths of coverage based on reference guided assemblies and estimated with samtools depth. (**a**) LHCA samples at different Cq values: Cq 20 (blue), Cq 22 (red), Cq 26 (gray), Cq 28 (yellow). (**b**) SGCA samples at different Cq values: Cq 20 (blue), Cq 22 (red).

Target enrichment efficiency was estimated by aligning trimmed and quality filtered reads to the *C*Las strain Psy62 reference genome and comparing alignment rate between enriched and non-enriched samples (Table 1). After trimming and filtering, 40–50% of the enriched reads were discarded due to insufficient read length and suspected probe contamination, while less than 5% of non-enriched reads were discarded (Table S3). Without enrichment, LHCA-20 and SGCA-20, the highest pathogen concentration samples, had genome coverage of 65 and 60%, respectively, both with 1x depth of coverage (Table 1). After SureSelect enrichment, both of these samples had 99% genome coverage with at least 250X depth of coverage. Enriched samples with the lowest pathogen concentration had 99% genome coverage and at least 70X sequence coverage. Only small portions of the genome were poorly covered, with more than 90% of the regions showing a depth of coverage of at least 20X across all samples (Fig. 1). In general, the same regions were not always missing, with only ~2 kb shared sites missing across samples. Of the seven shared sites missing across samples, four were in prophage regions that could reflect sequence diversity, and the remaining three regions only totaled approximately 200 bp. Pathogen DNA is enriched from 500- to 45,000-fold compared to non-enriched samples. All these results suggest that Agilent SureSelect XT HS target enrichment can effectively capture target DNA from complex *C*Las samples and significantly increase the pathogen DNA ratio.

**Table 1:** Alignment summary of *C*Las sample reads to the genome of *C*Las strain Psy62 using bowtie2.

ID	Enriched	Total Reads	Total Aligned Reads	Alignment Rate (%)	Fold Enrichment	Coverage (%)	Sequence Coverage
LHCA20	Yes	2,634,608	1,178,690	44.74	497	99.70	295X
	No	6,109,250	5,494	0.09		64.90	1.3X
LHCA22	Yes	3,486,268	2,121,784	60.86	4631	99.80	505X
	No	7,069,576	929	0.01		16.80	0.2X
LHCA26	Yes	3,299,660	691,607	20.96	45113	99.40	165X
	No	7,102,694	33	0.00		0.50	0X
LHCA28	Yes	3,277,008	328,121	10.01	19165	99.30	72X
	No	4,019,510	21	0.00		0.40	0X
SGCA20	Yes	1,467,124	1,089,745	74.28	1068	99.50	258X
	No	6,888,648	4,790	0.07		60.60	1.2X
SGCA22	Yes	3,093,380	2,302,812	74.44	4444	99.50	551X
	No	7,450,500	1,248	0.02		21.70	0.3X

### Prophage and genome diversity analysis

**Figure 2:**
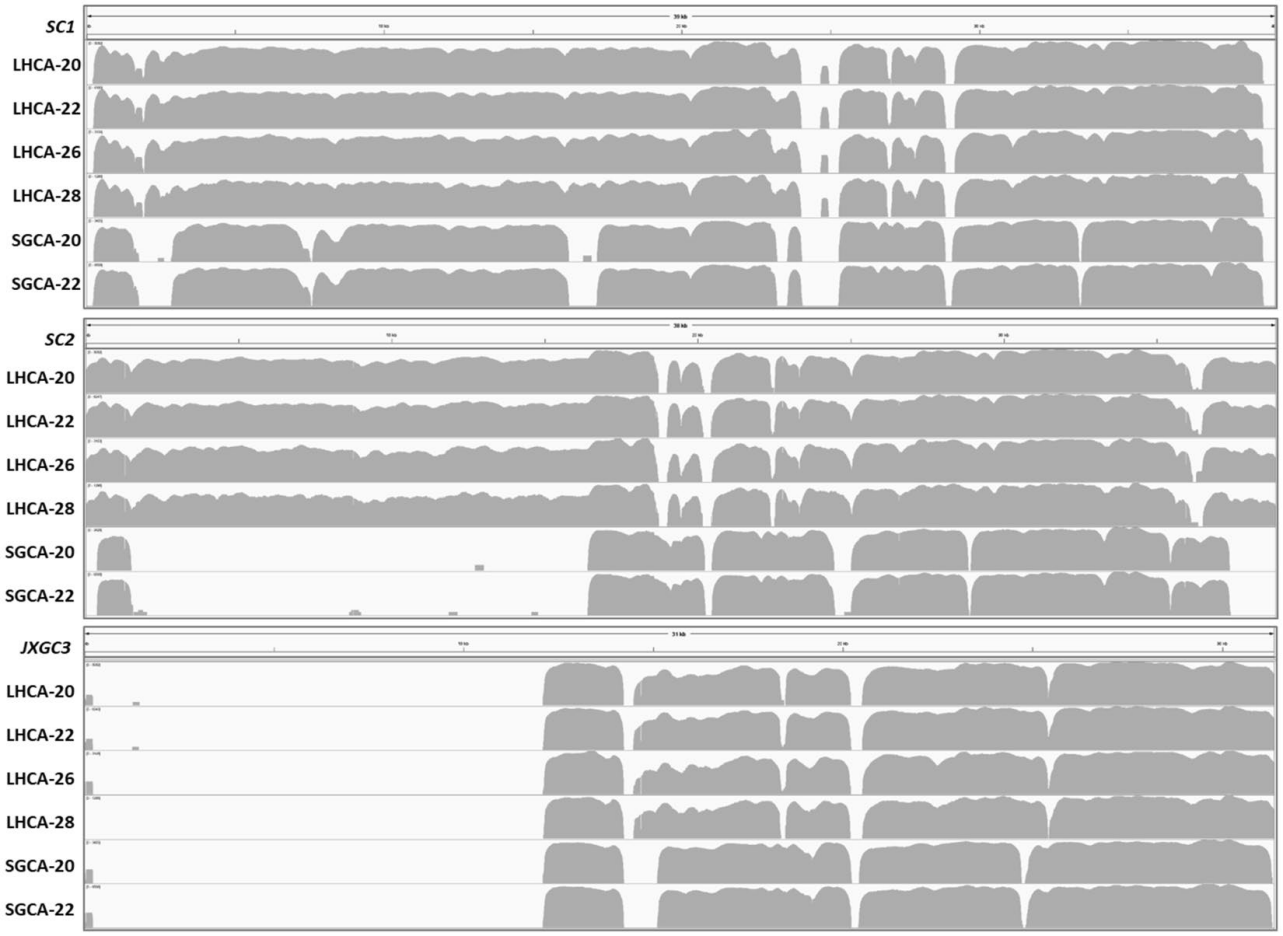
Profiles of *C*Las MiSeq reads mapping in reference to prophage SCI, SC2 and JXGC-3. Names of *C*Las samples were listed on the left. Reference prophage genome sequences were at the top. For each *C*Las samples, gray graphs represent read coverage in log scale. The alignment is generated using bowtie2 plugged in Geneious v 10.2.4, and visualized in Integrated Genome Viewer v2.4.10.

Next, we assessed how well enrichment captures the genome diversity of different strains. The most divergent region of the *C*Las genome is the prophage region, where strains can contain one to three prophages, with three prophage types known to date. For non-enriched samples, too few reads aligned to prophage reference sequences to estimate prophage type. Enriched samples, however, had enough reads to align samples to SC1, SC2 and JXGC3 prophage reference sequences. Each LHCA sample contained prophages SC1 and SC2, while SGCA samples contained only SC1 (Fig. 2). This pattern was consistent across different concentrations of the same strain.

**Figure 3:**
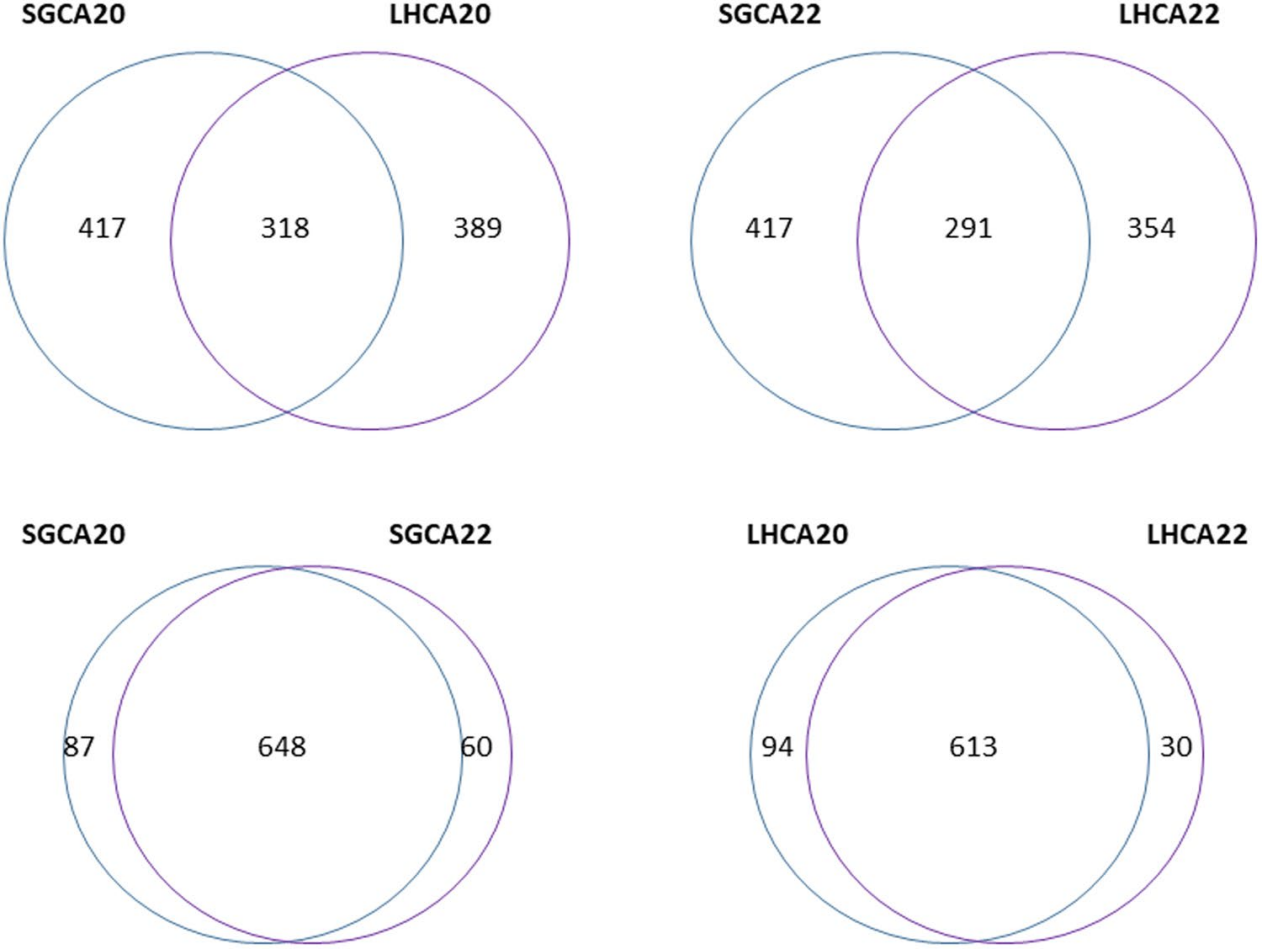
Venn diagrams show the overlapping of SNPs (single nucleotide polymorphisms) from different samples. The number in each circle represents the number of SNPs between the different comparisons. The overlapping number stands for the same SNPs identified between the different comparisons and the non-overlapping numbers specify the unique SNPs to each sample. SNPs were determined using Samtools v1.7.

To further analyze the repeatability and specificity of this method, we identified and compared the SNPs of these two strains at different Cq values. SNPs were determined based on the alignment profile to Psy62. More than 90% of SNPs were common between two high titer LHCA and SGCA samples, LHCA20/ LHCA22 and SGCA20/SGCA22 (Fig. 3 and Table S4). Less than 45% of SNPs in LHCA were identified in SGCA samples, suggesting this enrichment method does not change the pan-genome variability.

### Phylogenetic analysis

**Figure 4:**
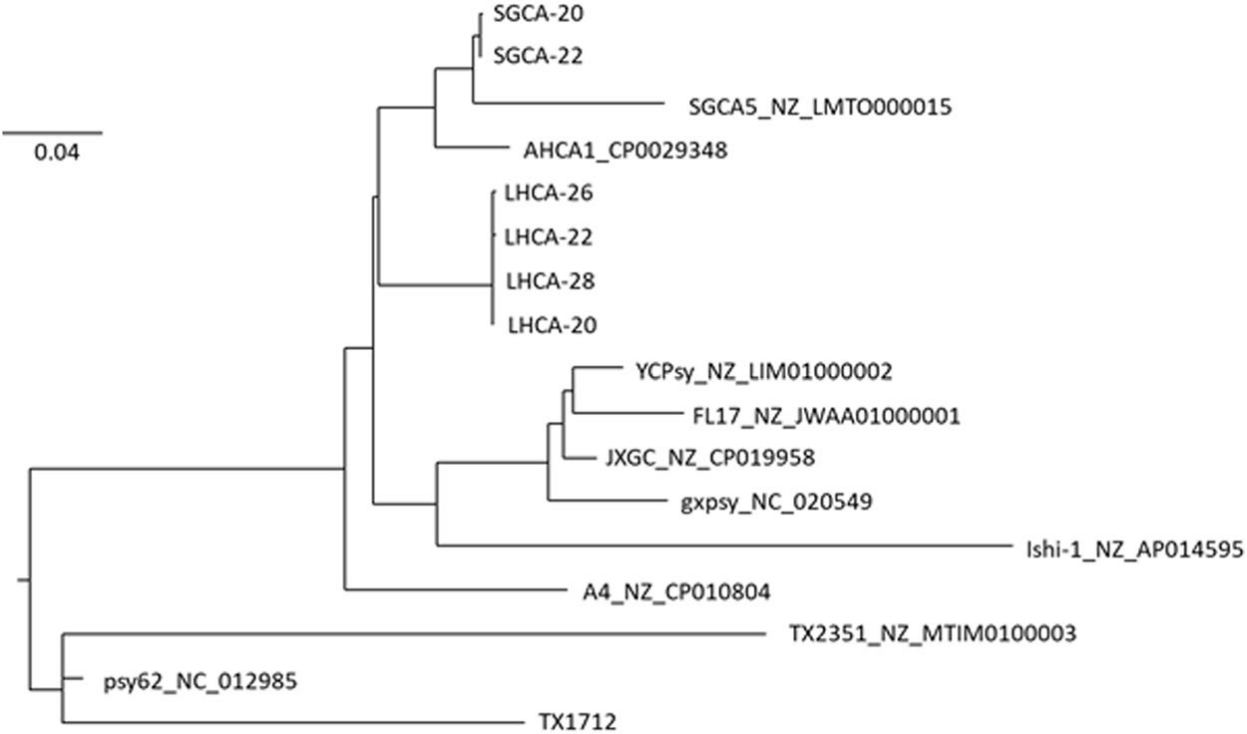
Phylogenic tree (ML midpoint rooted tree) of 849 core SNVs of “*Candidatus* Liberibacter asiaticus” strains generated with Rax Maximum Likelihood method. SGCA (20 and 22) and LHCA (26,22,28, and 20) were all sequenced in this study. All other genomes were obtained from NCBI. Trees were generated using RaxML v8.2.10 and visualized using FigTree v1.4.3.

**Figure 5:**
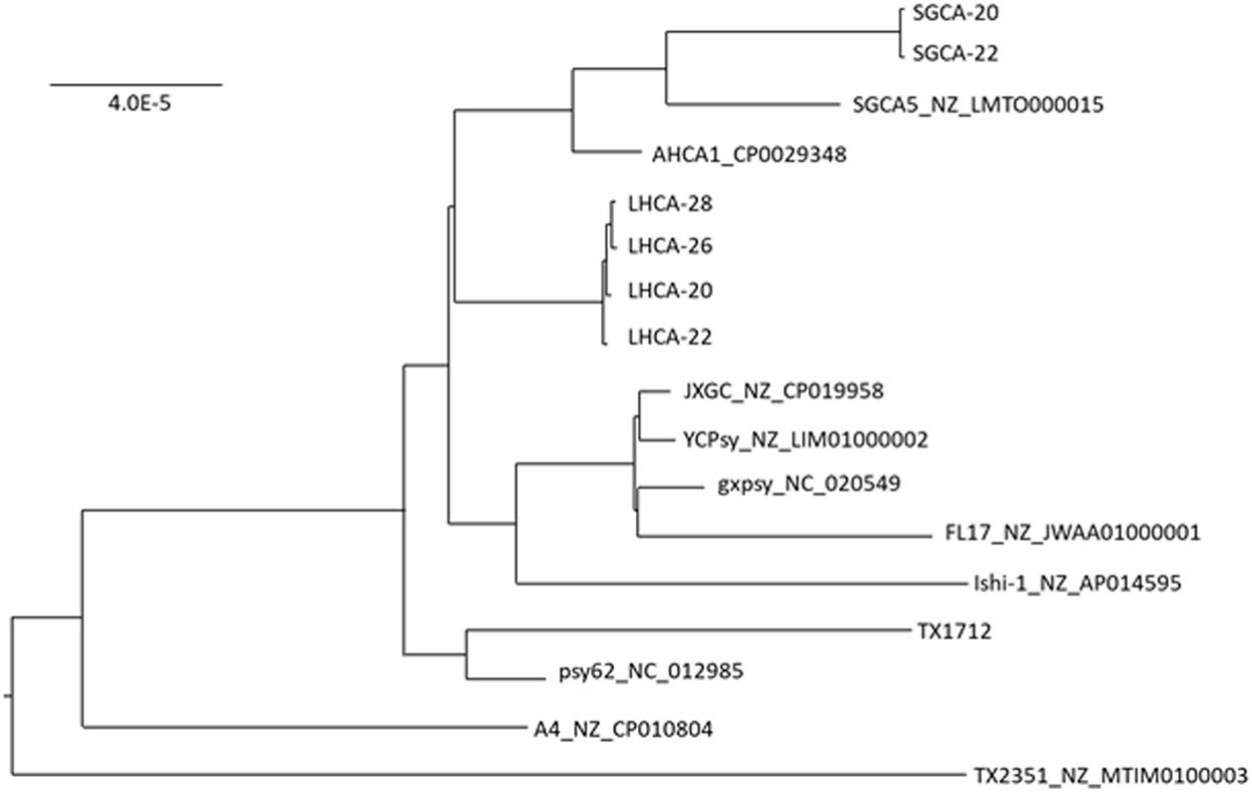
Phylogenic tree (ML midpoint rooted tree) of 935 core genes of “*Candidatus* Liberibacter asiaticus” strains, generated with Rax Maximum Likelihood method. All other genomes were obtained from NCBI. Trees were generated using RaxML v8.2.10 and visualized using FigTree v1.4.3.

We estimated phylogenies of all samples along with 11 available reference genomes, using both a SNP and pan-genome approach. The SNP tree clearly shows the separation of LHCA and SGCA strains (Figs. 4 and 5). The two SGCA strain samples are clustered together and most closely related to the previously reported SGCA strain, SGCA5. All four LHCA samples are also clustered together. The LHCA strain clusters most closely to the other reported California strains, AHCA1 and SGCA5, however it does form its own distinct clade from those strains too. The pan-genome phylogenetic tree based on core genes also demonstrates a similar branching pattern.

## Discussion

Over the past ten years, NGS (next generation sequencing) has been widely applied to identity pathogens, characterize genetic variants, and provide a molecular basis for building additional diagnostic tools. However, NGS technology has significant limitations when performing pathogen diagnostics in complex metagenomic samples. Without special enrichment, NGS can rarely detect low copy number pathogen sequences from complex samples due to low pathogen/host nucleic acid ratio. Prior to this work, obtaining a *C*Las whole genome sequence was a challenge. Nearly all draft genomes come from highly infected citrus or psyllids (usually with a Cq value lower than 23 using Li 16S qPCR), which limits strain diversity and epidemiology studies since not all samples can be sequenced reliably.

Researchers have used enrichment strategies to increase the number of target reads in sequencing. Previously, the NEBNext microbiome DNA enrichment kit coupled with the REPLI-g amplification kit was used to successfully sequence the HHCA genome from an infected lemon tree with 175 pg of *C*Las DNA per μl (roughly equivalent to Cq 23–24 using Li 16S qPCR^6^). This negative target subtraction coupled with microbial enrichment technique still required 78 million total reads to produce 10X genome coverage after assembly^24^. Hence, non-target enrichment of samples still makes *C*Las genome sequencing quite difficult and costly, and is not suitable for sequencing low titer samples (e.g. Li Cq 26 and above). The advantage to negative selection is it allows for the identification of new, large DNA insertions or mutations. Positive selection (like the SureSelect method described here) can enrich a target hundreds to thousands fold, making it possible to sequence low titer samples.

The positive enrichment approach described in this study shows a relatively simple and universal *C*Las genome enrichment method. We were able to efficiently get 99% coverage of the reference genome with over 70X sequence coverage using fewer than 5 million total reads even with a low to mid-titer pathogen sample (Cq value of 28.52). Thus this method makes large scale sequencing of the *C*Las genome more cost effective and applicable. More importantly, this method significantly pushes the sequencing limitation to much lower titer samples while preserving strain diversity. This is exemplified by the *C*Las genome of the lowest titer sample (equivalent to 28.52 Cq using Li 16S qPCR) being easily obtained with just 3.2 million total reads. Therefore, it could be possible to obtain the whole genome with even lower titer if more reads are used for the sample. Importantly, the RNA probe design of this positive capture method ensures retention of strain diversity, which other positive selection methods using primers run a risk of losing. This was exemplified by the phylogenetic analysis showing samples from two different locations clustering separately from one another (diversity retained), yet sequencing the same sample at different titer levels clustered together (reproducible results). These results indicate that this SureSelect target enrichment method can be used to sequence *C*Las more efficiently than the canonic NGS method. In the future, it will be interesting to determine the absolute sequencing limit of this method.

The most divergent region of the *C*Las genome is the prophage region, where strains can contain one to three prophages (or, in rare instances, none), with three known prophage types. Not surprisingly, we got the same prophage pattern for the SGCA strain sequenced in this study as SGCA5 (SC1 only), another strain from the same location^14^. Interestingly, LHCA contains both SC1 and SC2, meaning it has a different prophage profile and corresponds to the different clustering we observed in our phylogenetic analyses^18^ suggesting a potential different pathogen entry pathway. The probe set here use the SC1, SC2 and JXGC-3 as three prophage reference genomes, but we anticipate that it would capture all type 1, type 2 and type 3 prophage sequences if present in the samples. Although the mapping tracks show some different gaps among different strains suggesting uncovered non-conserved regions, the probes still capture sufficient prophage sequences for diversity analysis.

Besides the capability to sequencing medium to low titer samples, the total cost was also reduced by using SureSelect for the whole genome sequencing. Usually it costs at least $1500 to $3000dollars to whole genome sequence one high titer sample, but this was substantially reduced after using SureSelect target enrichment. In this study, it costs $500 per sample to obtain the whole genome, which includes $300 RNA probe per reaction and $200 sequencing price. The RNA probe price can drop further to around $100 dollar per sample if it is bulk order (96 reactions each order instead of 16).

In summary, our data suggest that SureSelect-based target enrichment system is an excellent and cost effective method for *C*Las whole genome sequencing from infected citrus samples, including those with pathogen titer far lower than those used in previous studies.

## Material and Methods

### Custom capture library design

The SureSelect custom capture library was designed by Agilent. Probes were designed for the capture of DNA sequences from the “*Candidatus* Liberibacter asiaticus” listed on Table S1 including whole genome sequences of Ishi strain (no prophage sequences), SC1 prophage, SC2 prophage, JXGC-3 prophage and unique sequences from the other five *C*Las strains with complete genomes available on NCBI. Overall, 12620 RNA probes were designed. Each probe consists of 120 mer RNA and the total probe size is 1.32Mbp (Table S1).

### Plant material and DNA extraction

Two *C*Las infected citrus branches containing LaHabra strain (LHCA) and San Gabriel strain (SGCA) were originally provided by California Department of Food and Agriculture (CDFA) and grafted to healthy citrus trees in the high containment green house of USDA APHIS PPQ Beltsville Laboratory. Successful grafted citrus trees were determined by HLBaspr real-time quantitative PCR from symptomatic leaves. *C*Las positive leaf samples from grafted trees were collected for genomic DNA extraction. Genomic DNA was extracted from petiole and leaf midrib tissue using the DNeasy Plant Mini Kit (Qiagen, Valencia, CA). The concentration of “*Ca*. Liberibacter asiaticus” was estimated using HLBaspr real-time quantitative PCR, giving a quantification threshold (Cq) value^6^. Four different Cq value (20.1, 22.84, 26.84, and 28.52) LHCA strain samples and two different Cq value (20.61 and 22.16) SGCA samples were selected to assess the sensitivity and selectivity of whole-genome enrichment and sequencing.

### SureSelect ^XT HS^ target enrichment: library preparation, hybridization and enrichment

A total of 1 μg input DNA per sample was used for SureSelect library preparation (Agilent, Santa Clara, CA). The library preparations were performed according to the SureSelect ^XT HS^ Target Enrichment System for Illumina Paired-End Multiplexed Sequencing Library protocol (Version A1, July 2017). The overall workflow is depicted in Fig. S1. First, all DNA samples were sheared using a M220 sonicator (Covaris, Woburn, MA) (duty factor 20%, peak/Displayed Power (W) 50 and 200 cycles/burst for 30 second duration time), and adaptors were ligated to end repaired DNA. Adapter-ligated libraries were purified using AMPure XP beads (Beckman Coulter, Inc., Brea, CA, USA), amplified, and then purified. Quality and quantity of libraries were determined by TapeStation using a D1000 ScreenTape (Agilent). Next, 1 μg of each library was hybridized with the SureSelect capture library. The hybridized libraries were purified with Dynabeads MyOne Streptavidin T1 magnetic beads (ThermoFisher Scientific, Waltham, MA), then the beads with captured DNA were washed one time with wash buffer 1 and five times with wash buffer 2 to remove non-specific binding. After all wash steps, the beads were suspended in 50 μl of nuclease free water. Twenty-five μl of the DNA libraries, bound to streptavidin beads, was amplified by PCR using SureSelect post capture primer mix and Herculase II Fusing DNA polymerase. The cycling conditions were as follows: 98 C for 2 min; followed by 16–24 cycles of 98C for 30 s, 60C for 30 s, and 72C for 1 min; and a final extension at 72C for 5 min., using 16 cycles for Cq 20 samples, 18 cycles for Cq 22 samples, and 24 cycles for Cq 26 and Cq 28 samples. After PCR, streptavidin beads were removed using a magnet stand, and the PCR products were further purified with AMPure XP beads. High quality libraries were identified with an Agilent TapeStation using High Sensitivity D 1000 ScreenTape and then pooled for sequencing.

### Illumina paired-end sequencing libraries preparation without target enrichment

We generated libraries for all six samples in parallel without enrichment using a TruSeq PCR free DNA library preparation kit (Illumina, San Diego, CA). A total of 2 μg input DNA was fragmented using a Covaris M220 with the same setting as SureSelect enrichment library preparation.

### Illumina sequencing

Sequencing of SureSelect enriched and non-enriched libraries was performed on an Illumina MiSeq platform (Illumina) on two separate v3 600-cycle cartridges (2 × 300 bp). Base calling and sample de-multiplexing were generated as paired FASTQ files for each sample. All raw read files were deposited to the SRA public database under BioProject ID PRJNA540608.

### Bioinformatics analysis

#### Read preprocessing

Raw reads were trimmed of adapter sequences and beginnings and ends trimmed where quality dropped to 0. Reads were discarded with a mean quality score of less than 10 or when shorter than 200 base pairs, to avoid potential probe contamination, using BBDuk v38.12 (http://bbtools.jgi.doe.gov).

#### Prophage diversity

To determine the prophage content of each sample, we aligned all the reads from enriched samples to SC1, SC2 and JXGC3 prophage reference sequences using bowtie2 plugged in Geneious v 10.2.4^25^, and visualized alignments in Integrated Genome Viewer v2.4.10^26,27^.

### Genome alignment and SNP calling

Filtered high quality reads were mapped to the HLB Psy62 strain reference genome (GenBank accession number GCA_000023765.2) using bowtie2 v2.3.3 in sensitive mode^23^. Optical and PCR duplicates were flagged in alignment files using Picard v.2.10.5 (http://broadinstitute.github.io/picard). Alignment files were filtered to remove PCR duplicates, retaining only reads in proper pairs with robust mapping quality (MAPQ ≥ 10) using Samtools v. 1.7^28^. The cleaned alignment files were used to call single nucleotide polymorphisms (SNPs) with Samtools using the mpileup function, and SNP and indel genotypes in Variant Call Formatted (VCF) format were generated using BCFtools v1.8^26^. VCF files were filtered to retain only variants sequenced to a minimum depth of coverage of 10 in enriched samples, and 3 in non-enriched samples. Shared and unique variants were compared within and between samples using vcftools “–diff-site” function.

#### Phylogenetic methods

Phylogenies were generated with all samples and 11 published genomes (Table S2) using two methods, ‘core SNPs’ and the ‘pan-genome’. Core SNPs were identified by mapping trimmed and filtered reads, as well as published genomes, against the Psy62 reference genome to create a whole genome alignment (including invariant sites), keeping sites with at least 10x coverage and greater than 90% consensus for each strain using Snippy v4.0 (https://github.com/tseemann/snippy). Genomic regions of high recombination were detected and removed with Gubbins v2.3.1^29^, and filtered polymorphic sites extracted to build phylogenies. A total of 849 core SNPs were used to construct 10 maximum likelihood trees using a general time reversible model with gamma correction (GTRGAMMA) and 10,000 rapid bootstraps with RaxML v8.2.10^30^. The tree with the highest likelihood across 10 runs was selected. The resulting tree was midpoint rooted and visualized using FigTree v1.4.3 (http://tree.bio.ed.ac.uk/software/figtree/).

For pan-genome generation, reads mapping to the Psy62 reference genome were extracted and assembled using SPAdes v3.12.0 with k-mer lengths of 21, 33, 55, 77, 99, and 127^31^. Contigs were reordered with Abacas v1.3.1^32^ using the *C*Las strain Psy62 as a reference, and then annotated with Prokka v1.12^33^. The annotated assemblies, as well as the 11 published genomes, were used to estimate the pan-genome with a 95% Blast ID cutoff using Roary v3.12.0^34^. Core alignments of 935 genes were extracted and used to estimate a maximum likelihood tree using RaxML, as outlined above.

## Supplementary information

Supplementary tables and figures
