# Supplementary material for: SureSelect targeted enrichment, a new cost effective method for the whole genome sequencing of Candidatus Liberibacter asiaticus
Source: Sci Rep. 2019 Dec 12;9:18962. doi: 10.1038/s41598-019-55144-4 (PMC6908597; doi:10.1038/s41598-019-55144-4)
Supplement: Supplementary file 1 — Supplementary tables and figures [file 41598_2019_55144_MOESM1_ESM.pdf]

Supplemental Information for:

SureSelect targeted enrichment, a new cost effective method for the whole genome sequencing of *Candidatus* Liberibacter asiaticus

Weili Cai[1,2], Schyler Nunziata[1], John Rascoe[1], and Michael J. Stulberg[1]*

[1]Science and Technology, Plant Protection and Quarantine, Animal and Plant Health Inspection Service, United States Department of Agriculture, Beltsville, Maryland, United States of America

[2]Department of Entomology and Plant Pathology, North Carolina State University, Raleigh, North Carolina, United States of America

***E-mail: Michael.Stulberg@usda.gov**

Supplemental

Table S1. ‘*Ca.* Liberibacter asiaticus’ sequences used for SureSelect target enrichment probe design.

Strain	RefSeq ID	Chr Start	Chr End
Ishi	NZ_AP014595_1	0	1190853
phage_JXGC3	KY661963	0	31449
phage_SC1	NC_019549	0	40048
phage_SC2	NC_019550	0	38997
Psy62	NC_012985_3	507161	507281
Psy62	NC_012985_3	983884	984004
Psy62	NC_012985_3	1081694	1081814
Psy62	NC_012985_3	1220451	1220571
gxpsy	NC_020549_1	492225	492345
gxpsy	NC_020549_1	945617	945857
gxpsy	NC_020549_1	1067993	1068113
gxpsy	NC_020549_1	1120798	1123438
gxpsy	NC_020549_1	1189164	1189644
gxpsy	NC_020549_1	1265215	1265455
A4	NZ_CP010804_1	200781	200901
A4	NZ_CP010804_1	206064	206184
A4	NZ_CP010804_1	206307	206427
A4	NZ_CP010804_1	366926	367046
A4	NZ_CP010804_1	500965	501085
A4	NZ_CP010804_1	583427	583787
A4	NZ_CP010804_1	673930	674170
A4	NZ_CP010804_1	716898	717018
A4	NZ_CP010804_1	796941	797181
A4	NZ_CP010804_1	1015512	1015992
A4	NZ_CP010804_1	1067840	1069760
A4	NZ_CP010804_1	1079582	1079702
A4	NZ_CP010804_1	1081228	1081348
A4	NZ_CP010804_1	1132389	1135029
A4	NZ_CP010804_1	1200996	1201356
A4	NZ_CP010804_1	1201567	1201687
A4	NZ_CP010804_1	1205167	1205287
A4	NZ_CP010804_1	1208766	1209366
A4	NZ_CP010804_1	1209596	1209836
A4	NZ_CP010804_1	1219099	1219459
A4	NZ_CP010804_1	1220511	1220631
A4	NZ_CP010804_1	1224181	1224781
A4	NZ_CP010804_1	1228154	1228394
A4	NZ_CP010804_1	1229114	1229234
A4	NZ_CP010804_1	1229701	1229821

JXGC	NZ_CP019958_1	500944	501064
JXGC	NZ_CP019958_1	649058	649178
JXGC	NZ_CP019958_1	796921	797161
JXGC	NZ_CP019958_1	1067812	1069732
JXGC	NZ_CP019958_1	1079554	1079674
JXGC	NZ_CP019958_1	1132370	1135010

Table S2. HLB genomes downloaded from GenBank and used in Phylogeny construction.

**Strain**	**Date Downloaded**	**Assembly**	**Level**
Pys62	5/8/2018	GCA_000023765.2	complete
Gxpsy	5/22/2018	GCA_000346595.1	complete
A4	5/22/2018	GCA_000590865.2	complete
Ishi-1	5/22/2018	GCA_000829355.1	complete
JXGC	5/22/2018	GCA_002216815.1	complete
FL17	5/22/2018	GCA_000820625.1	3 scaffolds
YCPsy	5/22/2018	GCA_001296945.1	9 scaffolds
SGCA5	5/22/2018	GCA_001430705.1	56 scaffolds
TX2351	5/22/2018	GCA_001969535.1	71 scaffolds
AHCA1	5/29/2018	GCA_003143875.1	1 scaffold
TX1712	7/16/2018	GCA_003160765.1	48 scaffolds
SGCA1*	5/29/2018	GCA_003149415.1	606 scaffolds
HHCA*	5/22/2018	GCA_000724755.2	239 scaffolds

Table S3. Summary of reads kept after BBDuk filtering.

**Sample ID**	**Cq Value (CLas)**	**Enrichment**	**Total Reads**	**After Filtering**	**Percentage Kept**
LHCA20	20.1	Yes	5,376,070	2,634,608	49.00%
		No	6,305,158	6,109,250	96.90%
LHCA22	22.84	Yes	5,600,568	3,486,268	62.20%
		No	7,302,226	7,069,576	96.80%
LHCA26	26.84	Yes	5,428,586	3,299,660	60.80%
		No	7,321,760	7,102,694	97.00%
LHCA28	28.52	Yes	5,801,194	3,277,008	56.50%
		No	4,155,700	4,019,510	96.70%
SGCA20	20.61	Yes	2,121,652	1,467,124	69.20%
		No	7,255,450	6,888,648	94.90%
SGCA22	22.16	Yes	4,928,054	3,093,380	62.80%
		No	7,719,964	7,450,500	96.50%

Table S4. Number of SNPs shared in particular combination of all samples as calculated using vcftools and --diff-site option. In parenthesis, number of SNPs unique to particular combination of samples as calculated using vcf-compare.

	LHCA20	LHCA22	LHCA26	LHCA28	**SGCA20**	**SGCA22**
LHCA20	707 (31)	-	-	-	-	-
LHCA22	613 (19)	645 (18)	-	-	-	-
LHCA26	618 (6)	576 (1)	654 (9)	-	-	-
LHCA28	589 (3)	550 (0)	588 (5)	624 (12)	-	-
SGCA20	318 (1)	284 (1)	308 (0)	295 (2)	735 (35)	-
SGCA22	297 (0)	291 (3)	290 (1)	269 (0)	648 (0)	708 (40)

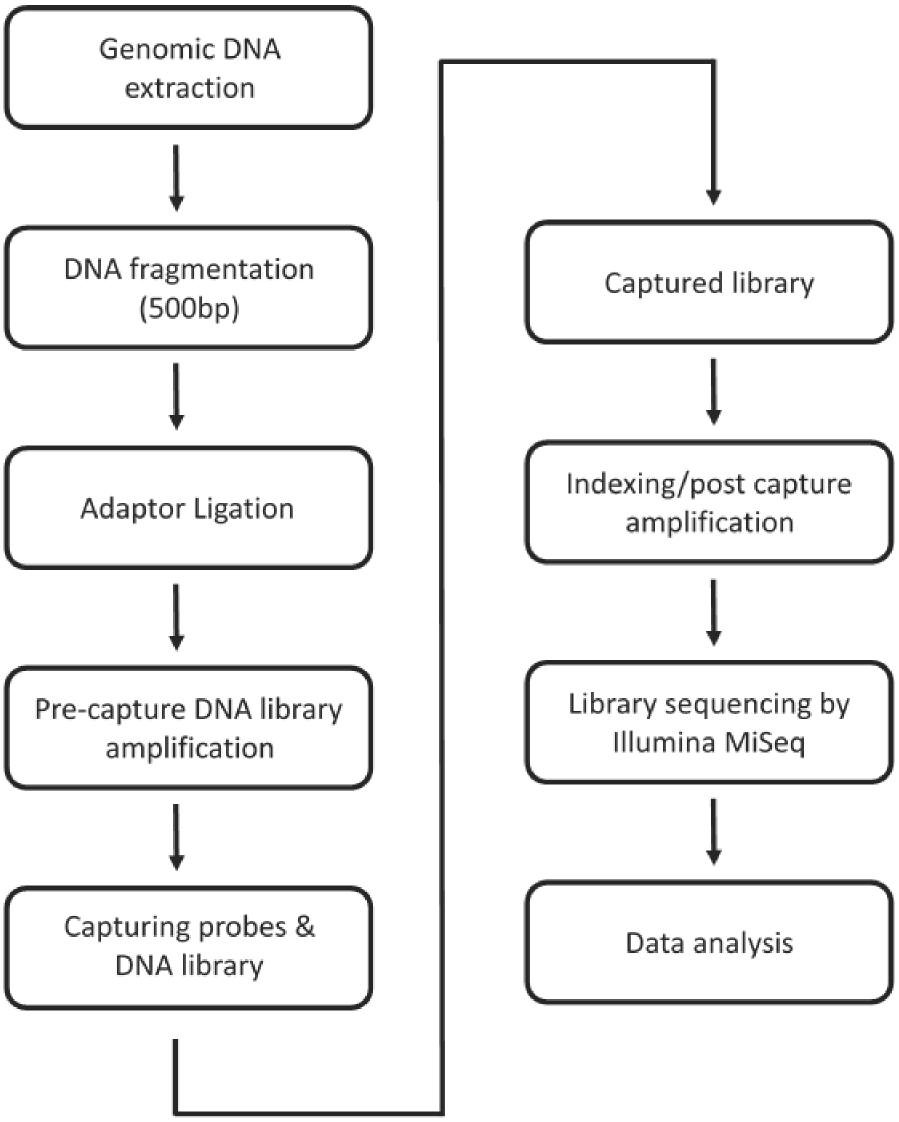

Figure S1. Work flow for SureSelect target enrichment of ‘*Ca.* Liberibacter asiaticus’.
